# Hyper-Reactivity to Salience Limits Social Interaction Among Infants Born Pre-term and Infant Siblings of Children With ASD

**DOI:** 10.3389/fpsyt.2021.646838

**Published:** 2021-05-14

**Authors:** Michal Zivan, Iris Morag, Jessica Yarmolovsky, Ronny Geva

**Affiliations:** ^1^The Gonda Multidisciplinary Brain Research Center, Bar Ilan University, Ramat Gan, Israel; ^2^Assaf Harofeh Medical Center, Zerifin, Israel; ^3^Sackler School of Medicine, Tel Aviv University, Tel Aviv, Israel; ^4^The Department of Psychology, Bar Ilan University, Ramat Gan, Israel

**Keywords:** infant behavior, gaze tracking, arousal, pupil diameter, social behavior, autism, prematurity

## Abstract

The ability to engage attention with selected stimuli is essential for infants to explore the world and process information relating to their surroundings. There are two main populations with a higher risk to develop attentional and social deficits whose deficits may arise from difficulties in regulating attention to salient cues: (1) siblings of children diagnosed with Autism; and (2) infants who were born pre-term. This study investigated infants' (*N* = 97) attention-engagement and pupil-dilation (PD) at 9 months of age, using a gaze-contingent paradigm and a structured social interaction. Specifically, we explored attention to stimuli with simple salient features (e.g., clear defined shapes, colors, and motions) vs. more complex non-social cues (amorphous shapes, colors, and motions) and social interaction in typically developing infants (TD, *N* = 25) and among two groups of infants at-risk to develop social difficulties (pre-terms, *N* = 56; siblings of children with Autism, *N* = 16). Findings show that the two risk groups preferred stimuli with simple features (*F* = 11.306, *p* < 0.001), accompanied by increased PD (*F* = 6.6, *p* < 0.001). Specifically, pre-term infants showed increased PD toward simple vs. complex stimuli (*p* < 0.001), while siblings showed a pervasive hyper-arousal to both simple and complex stimuli. Infants in the TD group preferred complex stimuli with no change in PD. Finally, the preference for the simple stimulus mediated the relationship between increased risk for social difficulties and decreased engagement duration in face-to-face interaction with the experimenter. Results suggest that activation of the attention-salience network shapes social abilities at infancy. Further, hyper-reactivity to salient stimuli limits social interaction among infants born pre-term and siblings of children with ASD.

## Introduction

The ability to engage attention with selected stimuli enables infants to explore the world and process information relating to their surroundings. Engagement is dependent upon arousal, a degree of alertness and vigilance during a wakeful state that enables reception and processing of data. Attention engagement during the neonatal period is positively related to the infant's arousal level (1–3). High arousal consists of increased physiological activation and responsiveness triggered by an event, action, object, or situation (4–6). Responding to exogenic stimuli is related to the phasic activity of the locus coeruleus norepinephrine (LC-NE) system (7). In infants, changes in arousal tend to precede subsequent changes in attention (2). Arousal responses may be expressed in multiple ways. Pupil dilation (PD) is a well-documented method to evaluate arousal responses (8–10). LC-NE activity is directly related to pupil dilation both among animals (11) and humans (12, 13), and thus pupil diameter recordings using eye-tracking equipment can be used to estimate phasic LC activity (14). Increases in pupil diameter reflect arousal in response to salient stimuli (9, 15, 16), complex cognitive processes [e.g., understanding other's emotions; (8) and unexpected sounds (10)]; and has been useful in evaluating reactivity in young infants (17). For example, increases in pupil dilations are evident in response to novelty and violations of infants' expectations (17).

Dynamic changes in arousal are thought to track the degree of uncertainty of the environment (2). In more uncertain environments, arousal is upregulated, leading to increased sensitivity to the surroundings and, in turn, to reductions in uncertainty (7, 18). Hence, hyper-engagement with arousing salient stimuli in the environment is seen in neonates (19). With maturation, exposure to a myriad of stimuli, and learning, the infant forms predictions. This in turn results in a differential effect, such that simple, predictable cues gradually cease to elicit augmented responses, while relatively complex ones gain more attention. Earlier work has shown that infants' visual preferences are controlled by an interaction between endogenous and exogenous arousal factors (2, 20–22). Effects are such that at 1-month-old infants prefer more arousing stimuli when less aroused (after feeding); and slower, less arousing stimuli when more aroused [before feeding or after exposure to highly arousing stimuli; (20)]. This relationship typically declines by 2–3 months of age (23).

The post-neonatal change, evident in typically developing infants, enables greater endogenous regulation of attention; associated with maturation of cortical networks, such as the frontal eye fields, prefrontal cortex, and anterior cingulate (24–26). These top-down processes of attention regulation and control promote the ability to direct attention voluntarily (endogenous factors) toward prioritized stimuli. Downregulation of the arousal response facilitates inhibiting engagement toward salient, yet repetitive exogenous stimuli that bear minimal new information (23, 27–29) and directing attention to more meaningful cues. As such, with the support of dynamic arousal changes, neonates manage to focus attention on relatively simple salient stimuli, while older infants prefer more intricate details that convey novel meaning (30–33).

There are two main populations with a higher risk to develop attentional and social deficits whose deficits may arise from difficulties in regulating attention to salient cues: (1) A genetic risk: Siblings of children diagnosed with ASD. Among this population 18.7% of infants will grow to develop ASD and 28% will grow to develop a broader Autism phenotype (34, 35). And (2) Prenatal exposure risk: Infants who were born pre-term. Among this cohort, 7–9.7% will grow to develop ASD (36–39) and many exhibit social-emotional difficulties (40). Notably, both groups are prone to hyper-arousal (41, 42). Increased susceptibility for ASD among the genetic risk group points to the possibility that they will be more profoundly affected by arousing cues than the prenatal exposure risk group. To date, these populations are typically studied separately (43–48) with the main focus on social attention. Fewer studies have explored visual attention in the presence of non-social stimuli (49). Among these studies, findings show relations between high rate stimulus preference and later ASD diagnosis among infants born pre-term (50, 51), and repetitive visual exploration of simple (high contrast spinning) objects among infant siblings who were later diagnosed with ASD (52). Given the domain-general framework of ASD and the postulation concerning an atypical interaction between arousal dynamics and attention in Autism, more research is needed in both risk populations, particularly in exploring arousal responses to simple non-social cues and their relations to social interaction early in development.

In recent years, longitudinal data concerning pre-maturity has uncovered that pre-term birth increases the susceptibility for an array of impairments in the social domain, evident from atypical gaze aversion noted at 4–6 months (43) peer problems and social withdrawal at childhood and overall lower social competence (53), as well as difficulty establishing relationships at adulthood (54). These deficits were thus far thought to be related to both exposure expectant and exposure dependent deficits given that the infants did not have sufficient opportunity to undergo necessary maturation *in utero*, and were exposed pre-maturely to extra-uterine cues (40); resulting in a wide-spread neural network alterations that mostly affect the social brain (40). Hypotheses regarding social deficits of individuals with Autism also shift from domain-specific to more domain-general. Social deficits in Autism Spectrum Disorders (ASD) result from widespread structural and functional impairments in the brain rather than a specific social brain network dysfunction. Similarly, involvement of the social network has been noted to infants born pre-term (40). Nevertheless, the study of infants at risk to develop social interaction difficulties, has mainly focused on social-communication behavior and responses to social stimuli (43–46, 48, 55) or social-emotional behavior (56). Resulting in a gap in the literature on attention toward salient non-social stimuli; stimuli that may elicit responses at infancy in ways that reflect a domain-general view of populations with social difficulties, such as people with social-communication disorders in its broad phenotype.

Support for the importance of non-social stimuli in ASD comes from data collected by subjective clinical observations, noting a preference for simple repetitive stimuli in young infants who later develop Autism (50) as well as in children with ASD (57, 58). This behavior represents one expression of the restricted and repetitive behavior characteristic of ASD (59). Hence, exploring hyper engagement and arousal to simple non-social repetitive stimuli in infants at higher risk to develop ASD and its broader phenotype offers a significant step in studying populations at risk for ASD early in development.

Individuals with ASD as well as infants at risk to develop social and communication deficits show atypical visual attention, including poor arousal regulation (60, 61). A study among neonatal intensive care unit graduates noted a relationship between abnormal arousal modulation at 4 months in infants born pre-term, evidenced by a preference for high-rate stimuli and ASD outcome (50). Other studies have reported attention orienting deficits with prolonged disengagement from a fixation stimulus to a peripheral one during the gap-overlap task (62, 63), difficulties disengaging attention from salient cues (48), and restricted focused attention and object exploration (52, 64), suggesting difficulty regulating attention and pre-occupation with simple geometric shapes among toddlers with ASD (46).

Although the literature dealing with early attentional regulations to non-social stimuli among infants at risk is limited particularly with regard to infants born pre-term, reported findings suggest abnormal engagement patterns among at-risk populations. Clinical observations of patients with ASD point to a comparable direction. One typical behavior among individuals with ASD is an unusual visual preference for simple high-rate salient repetitive stimuli, such as a moving fan blade or spinning wheels (52). This visual preference may result in hyper-focused visual attention toward such stimuli for long periods without disengaging (62, 65), while more complex and informative stimuli pass by unattended (66). Similar directions emerge using a computational model used to analyze eye-tracking data in response to free exploration of natural scene images. Such work suggests differences in stimuli saliency between adults with ASD compared to controls. Specifically, individuals with ASD show higher attention toward low-level saliency (e.g., contrast) compared to controls, whereas controls showed higher attention to stimuli with semantic-level saliency (e.g., interpretation of the image, such as a depiction of a jumping jack- for which salience emerges not by the presented cue features, but from the anticipation of a probable surprising event) (67). Taken together, these data suggest an atypical interaction between the arousal system and attention orienting in patients with Autism. With literature going as far as suggesting that the over selective attention and restricted behavior evident in Autism is a regulatory mechanism to cope with poor regulation of arousal, which is evident in individuals with Autism (68, 69). As such, probing this interaction between arousal dynamics and attention orienting may be a promising lead in evaluating young infants at risk to develop social-communication deficits.

Given the pervasive nature of the deficits noted in both groups; which may present more subtly in infants born pre-term and in a more varied form in populations with ASD; and the importance of early diagnosis to afford intervention while brain plasticity is at its best (70), the scientific community searches for objective behaviors that are detectable at infancy relating to the infant's social functioning. In keeping with optimizing development as efficiently as possible, we targeted attentional control, social orienting/engagement, positive affect, and communication (71). The current study, therefore, examined visual attention to non-social stimuli in young infants who are at increased risk of developing social difficulties due to pre-maturity or genetic risk. Further, following a pervasive domain-general developmental supposition we aimed to relate non-social visual preferences and pupillary responses with social engagement.

A few studies explored pupillary response in cognitive tasks with infants born pre-term. Most research thus far focused on the development of the pupillary reflex (72). One survey among 9-month-old infants at risk of developing ASD found increased pupil dilation in response to faces with emotional expressions compared with controls (73). Pupillometry studies among children and adolescents with ASD suggested that restricted behavior and narrow focus of interest are related to altered phasic LC-NE activity, leading to hyper-arousal (74, 75). Findings in toddlers and children with Autism show hyper-phasic LC-NE activation that leads to hyper-focused attention (74) and altered pupil dilation while watching scenes of human interactions (76–79). The authors suggested that an altered LC-NE activation mechanism among individuals with ASD underlies atypical attentional mechanisms leading to reduced social attention (80). However, very little is known about attraction and arousal in response to non-social cues, and its relation to social engagement. Integrating the available findings suggests that disruptions in the interaction between dynamic arousal changes and attention may signal difficulty in engagement early in development.

The current study thus aimed to examine the notion that infants who may be at higher risk of developing ASD or social deficits would experience hyper-arousal and differential engagement in response to high contrast simple stimuli, with a particular focus on infants born pre-term. Further, we explored the supposition that the expressed arousal to simple cues related to the infant's ability to sustain social engagements. A challenge in studying arousal behavior in young infants centers around avoiding forced choice, over-exposure and fatigue. However, operationalizing the current supposition was possible using gaze-contingent paradigms.

Paradigms measuring preferential looking provide valuable information in infants born pre-term (81) and in toddlers with ASD (46, 82, 83), yet classic preferential looking paradigms that require the infant to meet a familiarization or habituation criteria pose a risk of over-exposing infants to multiple arousing cues. The use of real-time gaze contingency enables the interactive use of eye-tracking, beyond its traditional recording properties. It creates a self-driven design in which the preferred stimulus is initiated and terminated by the infant. Infant-controlled exposure is particularly advantageous when exploring hyper-arousal, as it limits over-exposure, fatigue, and procedure-related hyper-arousal. Given these critical advantages, developing a gaze-contingent paradigm for exploring hyper-arousal is particularly vital for studying populations with an increased risk of developing ASD.

We aimed to explore a hyper-arousal framework that would account for attentional differences among high-risk infants and controls by studying gaze durations and pupil dilation in response to non-social straightforward content. This study, for the first time, allows comparison of engagement and arousal between siblings of children with ASD and infants born pre-term, under the same design, and investigates relations between non-social stimuli engagement and arousal and live social interactions. We expected that at the young age of 9-months, infants would prefer visually salient stimuli over stimuli with added semantic content, regardless of risk. Further, compared with controls, infants at risk were hypothesized to select simple featured repetitive stimuli and experience hyperarousal, measured by PD dilation, during stimuli watching compared to baseline and as compared to control infants. Given the notion that infants with a familial risk would be more profoundly affected than infants born pre-term, the siblings group is expected to show a general hyper-arousal effect. In contrast, the pre-term group would show more-selective hyper-arousal as a function of stimulus complexity. Finally, given the domain-general hypothesis, we postulated that preference for simple cues would limit social engagement behavior. We, therefore, expected relations between engagement with the simple stimuli may mediate the relationship between group and latency to disengage from direct social interaction.

## Materials and Methods

### Participants

Nine-month-old infants (*N* = 100, age corrected for pre-maturity) were recruited from the primary urban center of Israel. Neonates born pre-term (before 37 weeks' gestation) were recruited from the NICU at Sheba Medical Center during hospitalization after birth (pre-term group). Siblings of children diagnosed with ASD were recruited at the Israeli Society for Autistic Children (ALUT; Siblings group). Low-risk infants born at term served as controls. Exclusion criteria included severe complications after birth (e.g., cerebral hemorrhage) and hearing or vision deficits (*N* = 1 vision, pre-term; *N* = 2 significant developmental delay, control). The final study cohort consisted of 97 infants: 56 infants born pre-term (57.7%), 16 siblings of children diagnosed with ASD (16.5%), and 25 controls (25.7%; Table 1). No infants in the control group had siblings with ASD or were born pre-term, no infants in the siblings' group were born pre-term and no infants in the pre-term group had siblings with ASD.

**Table 1:** Demographic statistics as a function of risk group.

	**Controls**	**Pre-terms**	**Siblings**	***p***
*N*	25	56	16	
Gender (%Females)	40%	46.40%	43.75%	*p* = 0.864 (NS)
Gestation age (Weeks)	38.98 ± 1.095	30.35 ± 2.88	38.66 ± 1.23	*p* < 0.001 (*post-hoc*): *p* (P-C) <0.001 *p* (P-S) < 0.001 *p* (C-S) = 1(NS)
Birth weight (Grams)	3,312 ± 442	1,346 ± 446	3,145 ± 586	*p* < 0.001 (*post-hoc*): *p* (P-C) < 0.001 *p* (P-S) < 0.001 *p* (C-S) = 0.978(NS)
Test age (Months)	9.66 ± 0.77	9.70 ± 0.99	9.22 ± 0.95	*p* = 0.187(NS)

*P, Infants born pre-term; C, Control group; S, infant siblings of a child with ASD*.

This study was approved by the Institutional Review Board of Sheba Medical Center and the Ethics Committee of the Psychology Department at Bar-Ilan University. Parents signed informed consent before participation. A modest token of gratitude was given to the participating families irrespective of performance (value $20).

### Procedure

#### Eye-Tracking

Participants were tested individually in a comfortably illuminated (30 lux), quiet inner room, enclosed with a gray curtain. The illumination in the room was kept constant to avoid changes in pupil diameters due to light conditions. The experimenter monitored participants' gaze behavior on a separate external monitor. Participants were positioned in front of a computer, approximately 60 cm from the screen. Infants sat on their caregiver's lap or in a high-chair with the caregiver standing behind them. Caregivers were instructed to avert their eyes during stimuli presentation so that their gaze would not interfere. Gaze was monitored using a Tobii© 1750 eye tracker that tracks both eyes at a 50 Hz sampling rate, with a rated accuracy of 0.5 degrees. The task was programmed specifically for the current study and operated using E-PRIME-2.0 PRO©. Before data collection, a 5-point eye calibration was performed.

#### The Gaze Initiated Stimuli Preference Task

**Figure 1:**
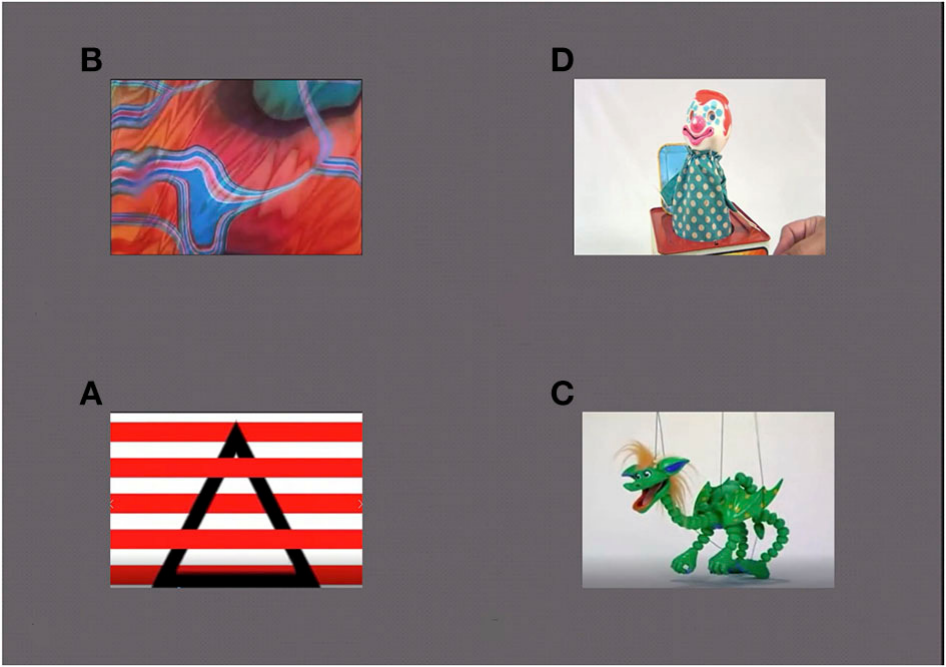
GISP task images. The bottom left panel represents the simple stimulus, the top left panel represents the complex stimulus and the two right panels represent the semantic stimuli.

A Gaze Initiated Stimuli Preference (GISP) free exploration task was designed using gaze contingency to observe gaze behavior in the presence of video stimuli with different complexity levels. Four images were presented on the screen, each displayed equidistant from the center (Figure 1). Fixating on an image activated each video, and shifting gaze from one image to the other terminated the current clip and started the new one. The session was complete after 1 min of total fixation time.

Of the four clips presented, the two clips presented on the left side of the screen attracted the great majority of the infants' attention. These two cues were defined by visual processing characteristics and they differed in their complexity. The bottom left cue represented the simple stimulus, presenting red and white broad stripes with a moving black triangle. It contained highly salient cues characterized by high contrast, well-defined, simple shapes, with three dominant colors and fast motion (6.93 deg/s) in one direction. The top left cue represented the complex stimulus, presenting a multi-colored, moving amorphous shape, with finer details, low contrasts, and slow-motion (0.477 deg/s). The two less attended clips on the right side of the screen were defined by semantic processing characteristics, which included more easily interpretable images depicted by moving puppets. To ensure illumination alone could not affect pupil dilation differences, illumination levels were designed to be comparable and measured at an eye-socket distance. There were no illumination differences between the stimuli.

Four rectangular areas of interest (AOIs) were defined corresponding to the four stimuli clips. For each participant, the total fixation durations per stimulus was defined as the total fixation duration spent at the corresponding AOI divided by the total fixation duration toward the task. Fixation durations were defined using an I-VT fixation filter (84). Additionally, mean Pupil dilation (PD) during the 100 ms before the stimulus appeared was calculated as a baseline for each participant. Stimuli dependent PDs were calculated per participant by calculating the average PD during stimulus observation (i.e., while the participant was fixating on the relevant AOI). These sensitive data files were available for 69 (18 controls, 40 pre-terms, 11 siblings) of the 97 infants. No demographic differences were noted between infants whose data files were available compared with those who were not {Gender: Data not available:43% female, Data available 45% female [$x_{\left(1,\;97\right)}^{2}$ = 0.346, *p* = 0.852, Crammer's V = 0.019]; Chronological age: Data not available 10.8 ± 1.9 months, Data available 10.9 ± 1.6 months [*t*_(2, 95)_ = −0.294, *p* = 0.792, Cohen's d = −0.059]; Corrected age: Data not available 9.5 ± 1.1 months, Data available 9.6 ± 0.9 months [*t*_(2, 95)_ = −0.548, *p* = 0.585, Cohen's d = −0.123]}.

#### Social Engagement Procedure

An adaptation of the Behavioral Responsiveness Paradigm was administered to a subsample of forty-five participants after the gaze-contingent paradigm was completed (85, 86). During this task infants sat in a high-chair while an experimenter, who was blind to the infant's risk group, engaged with the infant by presenting a series of 10 objects or interactions. Each object/interaction was presented for 20 s followed by a 10-s break. Objects were presented at a distance of approximately 40 cm from the participant's face or by touching the infant with the object when relevant (e.g., a comb used to gently brush the infant's hair). The different objects presented represented various levels of social stimuli. For the purposes of the current study two levels of social stimuli were analyzed: low social stimulus trials in which the experimenter spoke to the infant while holding an object (a small toy) in front of her face; and high social stimulus trials in which the experimenter directed speech to the infant in a direct face-to-face interaction. The dependent measure from the social engagement task was measured by calculating the ratio of the infant's time spent engaging toward the low vs. the high social stimuli. Thirty-two participants from the high-risk groups (25 in the pre-term group, 7 in the siblings group) and thirteen participants from the low-risk group completed this procedure. The rest of the participants did not attend to the social stimuli in the task, or did not complete this task due to fatigue. No demographic or gaze behavior differences were noted between infants who completed the SEP and those who did not (Table 2).

**Table 2:** Demographic and gaze behavior comparisons of infants who participated in the SEP and those who did not.

	**Infants who did not participate in the SEP**	**Infants who participated in the SEP**	***p***
*N*	52	45	
Gender (% Female)	44.2%	44.4%	*p* = 0.983 (NS)
Chronological test age (Months)	10.96 ± 1.8	10.78 ± 1.5	*p* = 0.599 (NS)
Corrected test age (Months)	9.64 ± 1.14	9.57 ± 0.70	*p* = 0.714 (NS)
FD simple	0.475 ± 0.350	0.358 ± 0.386	*p* = 0.094 (NS)
FD complex	0.306 ± 0.274	0.347 ± 0.332	*p* = 0.500 (NS)

*SEP, Social Engagement Procedure; FD, fixation duration; PD, pupil diameter*.

## Results

**Figure 2:**
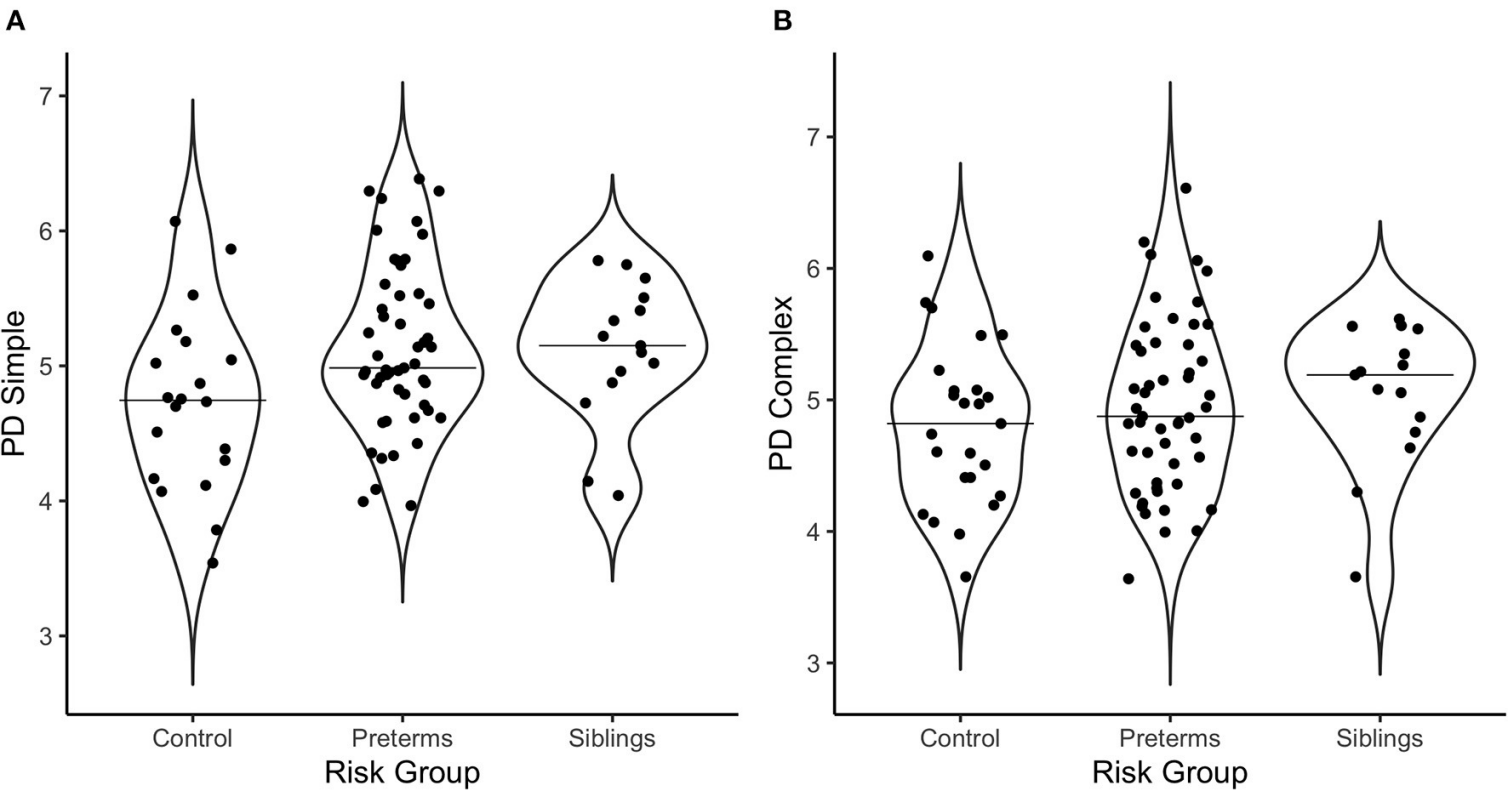
Violin plots depicting the distribution of pupil diameter in response to **(A)** the simple stimulus, and **(B)** the complex stimulus as a function of risk group. The line on the graph represents the median per group.

**Figure 3:**
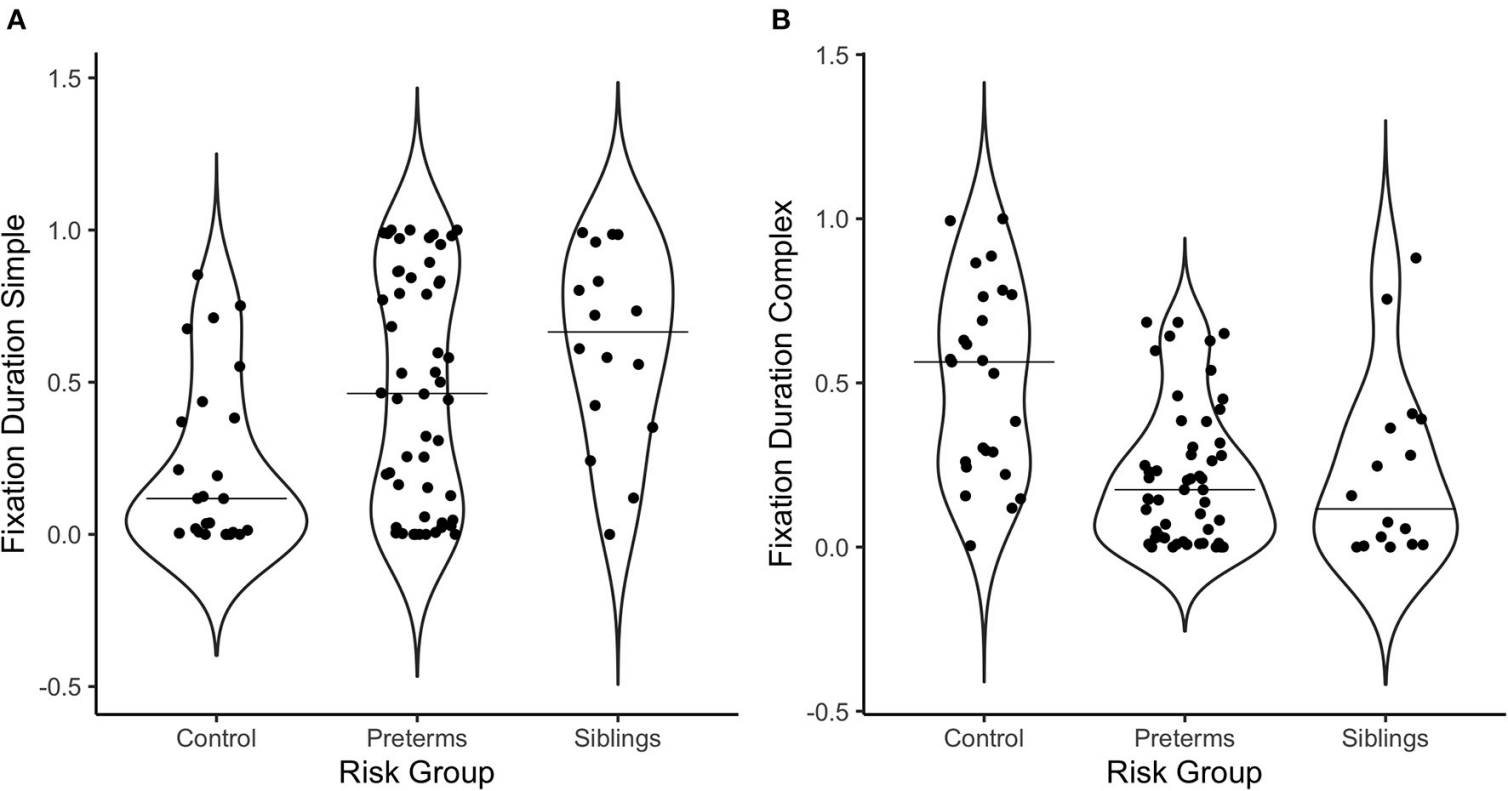
Violin plots depicting the distribution of fixation duration in response to **(A)** the simple stimulus, and **(B)** the complex stimulus as a function of risk group. The line on the graph represents the median per group.

Multivariate correlations and demographic information are presented in Table 3. Additionally, Figure 2 offers a visual depiction of the distribution of PD toward (a) simple and (b) complex stimuli as a function of risk group, and Figure 3 offers a visual depiction of the distribution of fixation duration toward (a) simple and (b) complex stimuli as a function of risk group. Additionally, participants with PD and fixation duration behaviors above and below the median behavior for the control group toward simple and complex stimuli were calculated. Distributions are such that 74.5% of the infants in the pre-term group and 80.0% of the siblings exhibited PD responses above that of the median for controls toward the simple stimulus [$x_{\left(2,\;86\right)}^{2}$ = 4.99, *p* = 0.08, Crammer's V = 0.241]; while 60.8% of the perterms and 73.3% of the siblings showed PD above the median for controls for the complex stimulus [$x_{\left(2,\;91\right)}^{2}$ = 1.79, *p* = 0.4, Crammer's V = 0.140].

**Table 3:** Correlation matrix and demographic information.

**Variable**	***M***	***SD***	**1**	**2**	**3**	**4**	**5**	**6**
1. FD simple	0.37	0.35						
2. FD complex	0.32	0.28	−0.53^**^					
3. PD simple	5.05	0.63	0.10	−0.25^*^				
4. PD complex	4.94	0.62	−0.02	−0.05	0.91^**^			
5. Corrected age (months)	9.58	0.91	−0.11	0.03	−0.17	−0.15		
6. Chronological age (months)	10.85	1.62	−0.00	−0.10	0.04	−0.02	0.69^**^	
7. Social engagement	4.37	4.11	0.42^**^	−0.27	−0.11	−0.14	−0.07	−0.08

*M and SD are used to represent mean and standard deviation, respectively*.

* *indicates p < 0.05*.

** *indicates p < 0.01; FD, fixation duration; PD, pupil diameter*.

Regarding fixation patterns, 73.1% of the pre-terms and 93.8% of the siblings exhibited fixation durations above the median for controls for the simple stimulus [$x_{\left(2,\;93\right)}^{2}$ = 10.3, *p* = 0.006, Crammer's V = 0.332]; while 11.5% of the pre-terms and 12.5% of the siblings showed fixation durations above the median for controls for the complex stimulus [$x_{\left(2,\;93\right)}^{2}$ = 14.2, *p* = 0.0008, Crammer's V = 0.391].

### Manipulation Check

In order to control for variance in age at recruitment, infants corrected age was included as a covariate in all analyses. To ensure that findings were not due to maturational effects of the pre-term group, all analyses were also checked with chronological age as a covariate, yielding no differences in significance outcome.

Compatible with the first hypothesis, a comparison of total fixation duration between the visual processing stimuli and the semantic stimuli (2 levels) as a function of group (3 levels: siblings, pre-terms, and controls) was conducted using a mixed design ANCOVA with corrected age as a covariate. Results revealed a stimulus type main effect [*F*_(1, 94)_ = 55.08, *p* < 0.001, partial η^2^ = 0.37], such that overall, infants fixated most of the time on the visually salient stimuli (M = 76.4 ± 3.6% SE) over the semantic stimuli (M = 23.6 ± 3.6% SE). No interaction of risk group and stimulus type was found, suggesting that at 9 months, regardless of risk, infants are still primarily focused on visual salience. Therefore, the subsequent analyses of gaze preference and autonomic reactivity concentrated on the two visual cues that attracted the most attention, the simple high salience, and the complex stimuli.

### Pupil Dilation Differences Between Groups as a Function of Simple and Complex Stimuli

**Figure 4:**
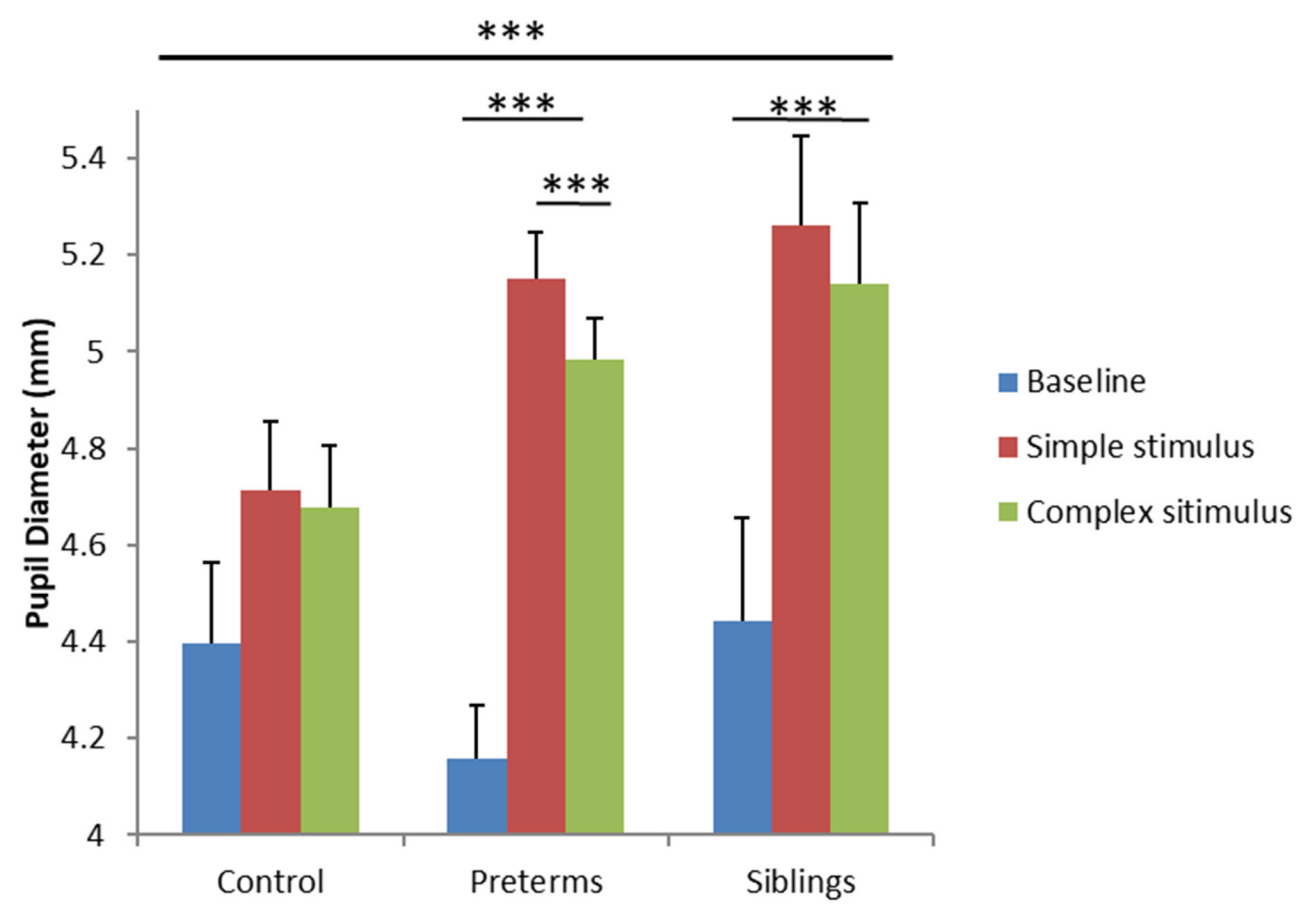
Pupil diameter during the GISP task as a function of task condition and risk group. ***p < 0.001.

To examine differences in arousal levels between high- vs. low-risk groups during the GISP task, a mixed design ANCOVA was run, exploring PD as a function of group (3 levels; control, infants born pre-term, infant siblings of children diagnosed with ASD) and task condition (3 levels; baseline-before stimuli onset, simple stimulus observation, complex stimulus observation), with corrected age as a covariate. Analysis yielded a task condition by group interaction [*F*_(4, 130)_ = 6.60, *p* < 0.001, partial η^2^ = 0.169; Figure 4]. *Post-hoc* comparisons showed that while the control group exhibited no PD differences across the three conditions, the sibling [*F*_(2, 64)_ = 11.207, *p* < 0.001, partial η^2^ = 0.259] and pre-term group [*F*_(2, 64)_ = 60.422, *p* < 0.001, partial η^2^ = 0.654] showed significant PD differences as a function of stimulus type. More specifically, both the sibling group and the pre-term group, but not controls, showed increased PD while observing both simple and complex stimuli compared to the baseline (*p* < 0.001 for all comparisons). In contrast, only the pre-term group showed larger PD toward the simple compared to complex stimuli (*p* < 0.001). As no group differences were seen between PD at baseline [control: M = 4.39, std = 0.66; pre-term group: M = 4.15, std = 0.75, sibling group: M = 4.46, std = 0.57; *F*_(2, 65)_ = 1.134, *p* = 0.328, partial η^2^ = 0.034], findings suggest hyper-arousal among the pre-term and the siblings groups while observing simple stimuli as compared with controls. Compatible with the second hypothesis, important differences were noted between the groups as a function of stimuli complexity. The pre-term group showed arousal selectivity between stimuli, characterized by larger PD when observing the simple compared with the complex stimulus. The siblings' group did not show selectivity in their arousal response to the different stimuli.

### Stimuli Preference Between Groups as a Function of Complex/Simple Stimuli

Outlier detection indicated that four participants from the pre-term group showed extreme fixation duration behavior, and were therefore removed from analyses. Descriptive analyses show that all groups spent close to 3/4 of their total fixation duration at their preferred stimulus, regardless of group (control 71.2 ± 1.5% SE; pre-term 74.6 ± 1.7% SE; sibling 74.6 ± 1.8% SE). A Bayesian ANOVA was conducted with percent of time fixating at the preferred stimulus as the dependent variable and risk group as the independent variable. Analysis yielded a BF_01_ = 6.88, percent error = 0.03. That is, it is 6.88 times more likely to accept the null hypothesis, indicating a moderately strong effect suggesting that preferred stimulus is likely not a differentiating factor between risk groups. Importantly though, the *type of preferred stimulus* was different among groups.

**Figure 5:**
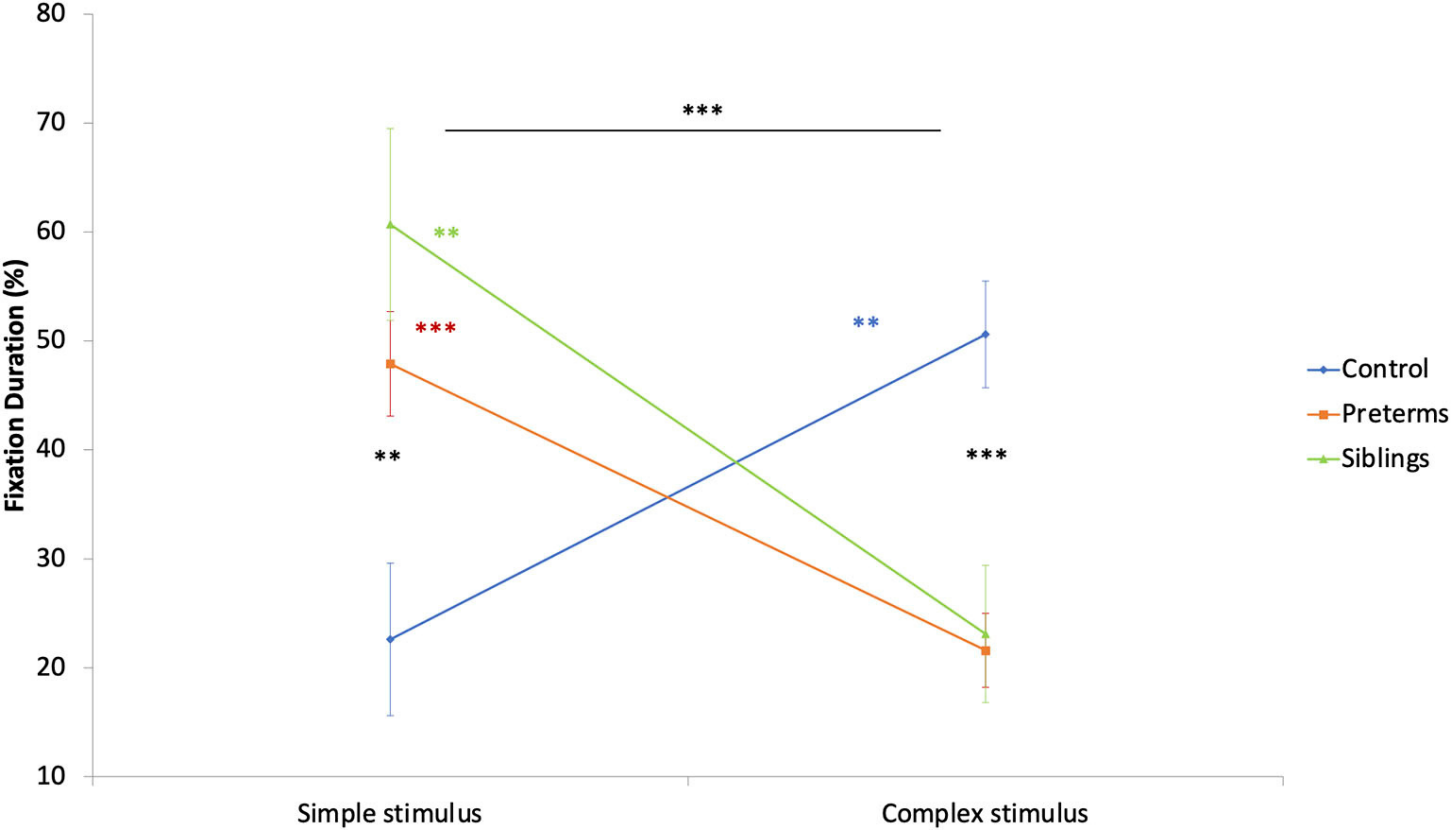
Fixation duration during the GISP task as a function of stimulus type and risk group. **p < 0.01, ***p < 0.001.

To explore group stimulus type preference effects, a mixed design ANCOVA was run. The total fixation duration toward stimuli (complex, simple), calculated as a percent of total fixation time at the task, was compared as a function of risk group, with corrected age entered as a covariate. A stimuli type by risk group interaction was found [*F*_(2, 89)_ = 11.306, *p* < 0.001, partial η^2^ = 0.203; Figure 5]. *Post-hoc* comparisons showed that infants in the control group had longer total fixation durations toward the complex compared to the simple stimulus [*F*_(1, 89)_ = 7.291, *p* = 0.008, partial η^2^ = 0.076], while the opposite was seen for siblings [*F*_(1, 89)_ = 8.196, *p* = 0.005, partial η^2^ = 0.084] and pre-term groups [*F*_(1, 89)_ = 13.357, *p* < 0.001, partial η^2^ = 0.130]. Bonferroni corrected comparisons showed differences between the groups in total fixation duration to the complex [*F*_(2, 89)_ = 12.258, *p* < 0.001, partial η^2^ = 0.216] and the simple stimuli [*F*_(2, 89)_ = 6.836, *p* = 0.002, partial η^2^ = 0.133], such that the pre-term and the siblings' groups looked longer at the simple stimulus than the controls. The controls looked longer at the complex stimulus than the high-risk infants. This suggests that despite being hyper-aroused while observing the simple cue, the high-risk groups maintained (or could not suppress) their engagement with simple cues.

### Relations Between Gaze Preferences and Social Engagement

Finally, to examine if simple stimulus preference (measured by the difference between FD simple minus FD complex) mediates the relationship between risk group and social engagement, with corrected age held as a covariate, a mediator model was conducted [(87), PROCESS Model 4]. For the purposes of the current analysis, siblings and pre-term groups were combined into one risk group and the variable was dummycoded such that 0 represents participants in the control group and 1 represents participants in the risk group.

**Figure 6:**
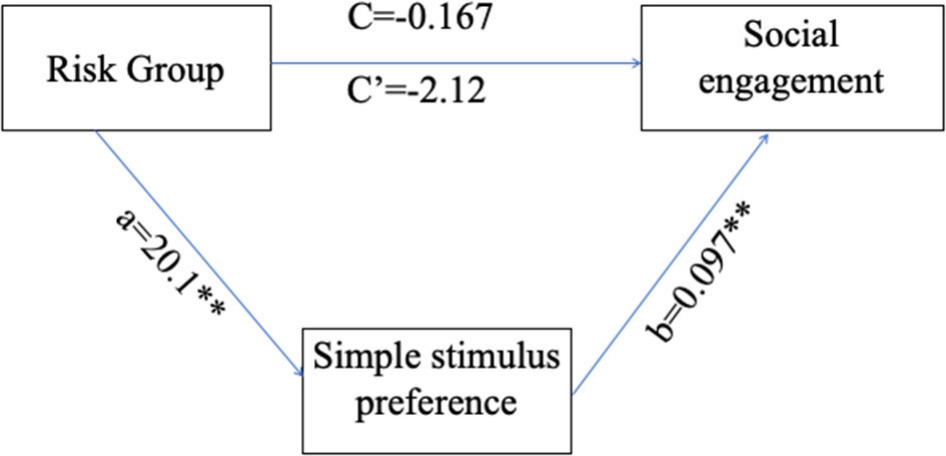
Simple stimulus preference as a mediator for the relations between risk group and low/high social stimulus engagement ratio. a, the path coefficient for the effect of risk group on simple stimulus preference; b, the path coefficient for the effect of simple stimulus preference on social engagement; c, the path coefficient for the direct effect of risk group on social engagement; c', the path coefficient for the total effect of risk group on social engagement. **p < 0.01.

Path coefficients for the PROCESS model (Table 4) and the overall regression model (Figure 6) revealed that the main effect of group on social engagement was insignificant (c' = −2.12, *p* = 0.1357). Further, simple stimulus preference offers a significant indirect path between risk group and social engagement (total indirect effect = 1.958, 95% 0.27–4.744), supporting a significant indirect-only mediation model (88). Overall, the model accounted for 24.23% of risk group variance. This model suggests that preference for the simple stimulus mediates the relationship between increased risk for social difficulties and decreased engagement duration in face-to-face interaction with the experimenter. Specifically, while risk is not directly related to social engagement, high risk for social deficits was related to preference for a simple over complex non-social stimulus, which in turn was related to engagement toward low rather than high social cues.

**Table 4:** Mediation model coefficients.

	**B**	**SE**	***B***	***p***
Path a	20.1	7.22	0.851	0.008
Path b	0.097	0.027	0.539	0.001
Path c'	−2.12	1.396	−0.497	0.136
Indirect effect	1.958	1.164	0.459	95% CI:0.27–4.744
Total *R*^2^	0.2423			0.0093

*Path coefficients a b and c' are presented as displayed in Figure 6; SE, standard error*.

Results show that hyper-engagement with simple non-social stimuli, more prevalent in risk groups, mediates the relations between risk group and social engagement.

## Discussion

Intrigued by the co-occurrence of attraction to simple, predictable cues and reduction in social engagement, we sought to explore whether infants with increased risk for social deficits are indeed more prone to hyper-engage with simple (non-social) signals while experiencing hyper-arousal even in self-guided gaze-contingent paradigms; and to study whether such preferences are related to the infant real-time social performance. Taking advantage of the advent of the self-guided paradigm, we were able to test the cues that attracted the infants most at 9 months of age. The data indicated that the infants were most interested in non-social simple and complex signals than cues that elicit interest based on their semantic value.

To date, studies with infants born pre-mature show less efficient and less mature attentional patterns (longer fixation duration, slower gaze shifting and lower novelty scores) among infants born pre-term compared to infants born full term during the first year of life (corrected age for pre-terms) (89, 90). The differences in attentional behavioral become more apparent as the pre-term infant develops into toddlerhood, and such differences were also found to be related to cognitive development in pre-school years (81, 91). Additionally, preference to highly arousing stimuli among infants born pre-term was found as a predictor for later emergence of ASD (50, 51). Prospective studies with infant siblings of children diagnosed with ASD provide compelling support for reduced or abnormal visual attention toward social stimuli (47, 73). The current study extends these data by focusing on visual preferences and reactivity in the presence of non-social stimuli.

We then proceeded to explore the degree to which stimuli complexity affects the infant's performance. Data shows hyper-arousal (increases in PD) among the pre-term and siblings' groups when observing simple stimuli compared with controls. These data support an altered interaction between arousal dynamics and attention orienting in cohorts at risk compared with controls. More specifically, while control infants no longer preferred simple cues and were not as aroused by them, infants in both risk groups preferred attending to the simple signal and experienced high arousal levels when viewing these cues. These non-social cues are compatible with previous reports with social faces (73). As such, current findings fill a literature gap that supports a social network deficit in pointing to a domain-general notion concerning complexity (irrespective of content) in directing the reactivity of infants in risk for social deficits.

Further, there was a gradient effect concerning arousal across risk groups. Both risk groups show hyper-arousal to both stimuli compared to the baseline condition, unlike the control group, which show no differences. However, results suggest differences in arousal selectivity between the pre-term and the sibling groups, with more preserved patterns among the pre-term group. Infants born pre-term show higher arousal to the simple stimulus than the complex stimulus. The sibling group, on the other hand, showed a generalized hyper-arousal response compared to the other groups.

These results support previous literature, suggesting altered LC-NE activity among individuals with ASD. Individuals with ASD tend to exhibit higher plasma NE (92) along with atypical pupil dilations in visual search tasks (74) and in response to social stimuli (93). Similarly, such trends were reported for infants at risk in response to social cues (73). The current study extends previous literature with new evidence regarding increased pupil dilation in response to non-social stimuli among infants at risk and differences in pupil dilations between different populations at risk, underscoring involvement of a similar mechanism in participants in both risk groups. These results may point to an atypical arousal regulation development among populations at risk (with a more preserved pattern among pre-term vs. sibling groups) and a preference for simple stimuli among increased risk groups, opposite to preferences seen in the control group.

The current study underscores the role of orienting to salient cues in social behavior; and relates the restricted visual attention pattern seen in patients with Autism to social engagement behavior. Importantly, current results uncovered a mediation relationship of the attentional grip by simple stimuli on the relationship between risk group and social engagement behavior. Our findings indicate that the tendency to engage with simple cues over complex ones mediated the relationship between risk group and social engagement behavior. Directions were indirect. That is, while risk was not directly related to social engagement; high risk for social deficits was related to preference for a simple over complex non-social stimulus, which in turn was related to engagement toward low rather than high social cues. This finding bears an optimistic outlook since the degree of attraction to simple signals may be an accessible target for intervention.

These data also deepen the understanding of behavioral reports in the pre-term infant population and infant sibling of children with ASD population. Pre-term infants show frequent short gazes and multiple gaze breaks during face-to-face interaction (44, 86) and more gaze aversions in a social context (43). Similarly, children with ASD and sibling infants show poor eye contact as an early characteristic of ASD (45, 48). Current findings suggest that difficulty regulating hyper-engagement in response to simple cues may be related to maintaining social engagement. These data are compatible with the notion that earlier maturing functions, such as hyper-engagement with simple stimuli, may limit social engagement (94–96).

The differences observed in attentional behavior among pre-term and sibling groups compared to controls concerning the involvement of the salience network point to a mechanism enabling a more significant impact for exogenous factors in the environment acting in both risk groups, with less influence of endogenous factors. It is evident, in particular, in increased PD among the high risk groups while observing the simple cue. This simple cue captivation expresses an altered regulation mechanism that limits the capacity of disengaging from a salient signal in favor of a more complex one. A previous study focused on movement signatures of infants at risk for ASD (97). Findings suggested the possibility of altered functioning of the top-down processes causing infants at higher risk for social difficulties to present a restricted behavior that reduces their sensitivity to the information from their surroundings. The current study results support the altered functioning of the top-down processes notion, focusing on disruption in the interaction between arousal responses and visual attention orienting to salient cues among 9 months old infants.

Current findings concerning self-regulation of autonomic reactivity add support to theoretical neurobiological models of infant self-regulation suggested by our group (96) concerning the primary role served by neonatal brainstem pathways in enabling self-regulation of reactivity and social attention (98). The model indicates that prenatal development of neural networks that facilitate arousal regulation affects emotional and attentional regulation. This line is also compatible with evidence from animal models of ASD and studies on humans with ASD, which suggests the involvement of the brainstem in the development of Autism (99). Integrating current results concerning hyper-arousal and preference of simple stimuli may suggest that hyper-activation of the salience network may be involved in the development of socio-emotional behavioral characteristics. Early occurring in the fetal period, hyper-activation of the salience network possibly elicits pervasive attention and learning deficits that further shape the development of multiple social and communication domains (100).

More specifically, current data provide behavioral support for the involvement of the salience network so far seen in older children using a neuroimaging study that examined the connectivity of large-scale brain networks between 7 and 12-year-old children with ASD compared to controls. This neuroimaging study pointed to hyper-connectivity in the salience network among children with ASD compared to controls (101, 102). The current results support the involvement of changes in reactivity to salience already in the first year of life, in a subtle form in infants born pre-term and a more pervasive form in infants who have siblings with ASD. Current findings show that these reactivity responses are related to social functioning. Taken together, findings point to a general notion whereby activation of the attention-salience network is involved in shaping social abilities at infancy. Further, hyper-reactivity to salience limits social interaction among infants born pre-term and siblings of children with ASD.

The main limitation of the current study is the smaller size of the siblings' group as compared with the pre-term group. The relatively small number of siblings is related to the known lower propensity of this group as compared with infants born pre-term in the general population, a limitation that is quite common in studies of siblings of children with Autism (103–105). The pre-term group was recruited directly from the NICU during hospitalization. It is important to bear in mind that pre-maturity is a heterogenous group in causes and outcomes, and therefore a larger sample size was important to take account for intergroup differences. Future studies may explore involvement of brainstem integrity in this group as well as other heterogenous factors to further understand effects. The third limitation of the study was the fairly significant loss of SEP data due to participants' fatigue. While we examined and found no demographic differences or fixation duration behavioral differences for those infants who participated in the social engagement procedure and those infants who did not, future studies may enable further exploration of SEP behaviors. Finally, the participants in the current study are younger than the typical diagnosis age of social deficits and ASD. This is one of its primary strengths, yet, we cannot yet suggest the current measures as early markers for persistent social difficulties without further longitudinal follow-up.

Overall, the domain-general salience network hypothesis regarding the emergence of ASD and social deficits suggests further investigation of autonomic reactivity in longitudinal studies of these populations may deepen the understanding of the mechanisms underlying socio-emotional difficulties and Autistic traits.

The current study also points to the advantages of using the gaze-contingent methodology in conducting experiments with young infants or challenged participants, especially when studying arousal and when exploring features of Autism. In the current study, we emphasized the usefulness of the gaze-contingent methodology, which enables the intuitive use of gaze to promote task progress, allowing very young participants to control the task with their eye at their own pace while avoiding over-exposure to undesired cues and fatigue (106–108). This advantage is especially important when studying arousal. Applying infant-controlled exposure to the stimuli may strengthen the assumption that the hyper-arousal shown is not driven by over-exposure, but rather by under-regulation of behavior in response to augmented arousal in the high-risk groups. Given the advents of the gaze contingency method in initiating exposure only upon participants' gaze direction to the selected cue, arousal is kept under participant control, to guaranty maximal participant comfort. Current results with this method show promise in further applications in the study of infants at risk for ASD and other social and neurodevelopmental disorders who struggle with arousal regulation issues in processing information and engaging in dynamic inter-personal interactions.

## Data Availability Statement

The datasets presented in this study can be found in online repositories. The names of the repository/repositories and accession number(s) can be found below: http://dx.doi.org/10.17632/jy9sg9s4wn.1.

## Ethics Statement

The studies involving human participants were reviewed and approved by the Institutional Review Board at Sheba Medical Center and the Ethics Committee at Bar Ilan University.

## Author Contributions

MZ and RG conceptualized and wrote the manuscript. IM, RG and JY co-authored the article and provided input on the final version. All authors contributed to the article and approved the submitted manuscript.

## Conflict of Interest

The authors declare that the research was conducted in the absence of any commercial or financial relationships that could be construed as a potential conflict of interest.
